# Stress Factors for the Paediatric and Adult Palliative Care Multidisciplinary Team and Workplace Wellbeing Solutions

**DOI:** 10.3390/healthcare12090868

**Published:** 2024-04-23

**Authors:** Maria Valentina Popa, Dana Elena Mîndru, Mihaela Hizanu (Dumitrache), Irina Luciana Gurzu, Dana Teodora Anton-Păduraru, Violeta Ștreangă, Bogdan Gurzu, Cristian Guțu, Eva Maria Elkan, Letiția Doina Duceac

**Affiliations:** 1 Faculty of Medicine and Pharmacy, Doctoral School of Biomedical Sciences, “Dunărea de Jos” University of Galați, 47 Domnească Street, RO-800008 Galați, Romania; maria_valentina_popa@yahoo.com (M.V.P.); mh202@student.ugal.ro (M.H.); 2 Department of Pediatrics, Faculty of Medicine, “Gr. T. Popa” University of Medicine and Pharmacy, RO-700115 Iasi, Romania; dana.anton@umfiasi.ro (D.T.A.-P.); streangavioleta@yahoo.com (V.Ș.); 3 Department of Preventive Medicine and Interdisciplinarity, Discipline of Occupational Health, “Gr. T. Popa” University of Medicine and Pharmacy, RO-700115 Iasi, Romania; irina-luciana.gurzu@umfiasi.ro; 4 Department of Morfofunctional Sciences, Faculty of Medicine, “Gr. T. Popa” University of Medicine and Pharmacy, RO-700115 Iasi, Romania; bgurzu@yahoo.com; 5 Clinical Medical Department, Faculty of Medicine and Pharmacy, ”Dunărea de Jos” University of Galați, 47 Domnească Street, RO-800008 Galați, Romania; cristian.gutu@ugal.ro; 6 Department of Morfofunctional Sciences, Faculty of Medicine and Pharmacy, “Dunărea de Jos” University of Galați, 47 Domnească Street, RO-800008 Galați, Romania; eva.elkan@ugal.ro; 7 Medical Department, Faculty of Medicine and Pharmacy, “Dunărea de Jos” University of Galați, 47 Domnească Street, RO-800008 Galați, Romania; letimedr@yahoo.com

**Keywords:** stress, paediatric palliative care, healthcare workers, pandemic, organisational policies, holistic approach

## Abstract

Background: Palliative care is a challenging specialty, especially when it comes to caring for children with serious life-limiting conditions and supporting their families. Workers face significant challenges and experience major impacts on their wellbeing. We conducted a qualitative study to understand the sources of stress in the palliative care team, their work expectations, and how they can cope with the demands. Methods: We used an online questionnaire about the causes of stress, the impact of the COVID-19 pandemic and the ways in which support is needed in the workplace. Results: Of the 56 palliative care professionals who participated in the survey, 57.1% considered the main causes of stress to be high workload, difficult emotional burdens (55.4%) affecting their outlook on life (61.2%), the death of patients (46.4%), and communication with patients’ families (26.8%). The COVID-19 pandemic increased stress levels for the majority of respondents (89.3%). The need for specialised training (53.6%), support groups, psychological counselling and adapted organisational policies was highlighted. Conclusions: The study demonstrates the importance of understanding the needs of both paediatric and adult palliative care staff in order to provide optimal care and support their balance in this demanding area of the healthcare system.

## 1. Introduction

According to the World Health Organization (WHO), palliative care is defined as “the prevention and relief of suffering for adult and paediatric patients and families facing problems associated with life-limiting illness” [1]. Paediatric palliative care is a specialised field that focuses on the compassionate and holistic care of children with life-limiting illnesses and their families [2]. Each year, more than 8 million children worldwide require specialised palliative care [3]. Burnout among staff working in paediatric palliative care units is a significant health challenge as this specialised area of care involves providing support and comfort to children with life-limiting illnesses and their families, which can be emotionally and physically demanding [4].

There are several factors that can make working in paediatric palliative care more stressful than working in adult palliative care. Although there are similarities in the principles of adult and paediatric palliative care, there are notable differences between the two age groups. In 2022, Buang et al. highlighted the key differences between paediatric and adult palliative care patients, which lie in patient characteristics, underlying diagnosis and comorbidities, and the duration of palliative care required [5].

While the age range for adults tends to be narrower, with the majority of patients aged 80 years and over [6], professionals working in palliative care services are dealing with a more diverse population of children [7]. Healthcare professionals need to adapt their approach to the child’s developmental stage and provide age-appropriate information and support as children are at different stages of physical, cognitive, and emotional development, which affects their understanding of illness, decision-making capacity and ability to communicate their needs [8]. In addition, children may have limited verbal communication skills, especially in younger age groups. To effectively assess and address the child’s needs and preferences, the palliative care team should use alternative methods of communication, such as visual aids, play therapy, or the involvement of a child life specialist [9]. Children need age-appropriate explanations of their illness and treatment, taking into account their cognitive abilities and emotional maturity. According to Palazzi et al., communication goals depend on the age group. For example, in infants, soothing and relieving distress and demonstrating care through a gentle touch are recommended, whereas in toddlers, additional interventions should be aimed at identifying distressing symptoms and validating emotional experiences [10]. In school-aged children, healthcare professionals are encouraged to obtain the information needed for diagnosis and treatment, encourage cooperation and compliance with recommendations, provide education about the illness, and demonstrate respect for individual choice and voice [10]. For adolescents, in addition to the goals for younger children, it is important to discuss goals of care and medical decision making and to discern hopes, concerns and fears [10].

Another important difference between children and adults is the underlying diagnosis and comorbidities. While in adults, cancer is the most common underlying diagnosis in palliative care networks [11], in children, a wide variety of congenital and acquired diseases and an increasing number of complex chronic diseases have been reported in the last few decades [12]. In addition to malignancies, the most commonly reported conditions in children requiring palliative care include the following: primary central nervous system disorders, biochemical and metabolic disorders, neuromuscular disorders, cardiac and pulmonary disorders, infectious and immunological diseases, and multi-organ disorders due to chromosomal aneuploidy or genetic defects [12]. As these wide-ranging conditions often have different disease courses, prognoses, and symptom profiles than adult diseases, palliative care interventions should be tailored to the specific needs and challenges. Understanding complex medical conditions and treatments for rare or life-limiting illnesses requires specialised knowledge and skills to manage them effectively.

The interdisciplinary nature of palliative care is also important. Paediatric palliative care teams often include professionals from a variety of disciplines, such as paediatricians, nurses, psychologists, social workers, and educators [4]. This collaborative approach addresses the unique needs of children, including their cognitive development, emotional wellbeing, and educational needs.

The family plays a key role in decision making and support. While adult palliative care focuses on the patient and involves family members primarily as decision makers acting as “surrogates”, paediatric palliative care focuses on the child and the family, with the family actively participating as both subject and object of the process [13]. Consequently, health professionals should consider the dynamics of the family unit, including parental preferences, sibling involvement, and the emotional impact on caregivers while providing comprehensive care. Paediatric palliative care often requires close collaboration with families. Building relationships with parents and other caregivers, providing emotional support, and helping them make difficult decisions can be emotionally demanding. The care team may also need to address the concerns of siblings and extended family members. In addition, the loss of a child can be particularly distressing for paediatric palliative care providers. Despite their best efforts, some children do not survive their illness, and providers may experience grief and a sense of personal loss. Witnessing the suffering of young patients and their families can be deeply distressing and have a profound impact on healthcare providers [14].

In addition, ethical dilemmas in paediatric palliative care may involve decision making for minors, balancing the best interests of the child with parental autonomy, and dealing with end-of-life decisions. Healthcare professionals must navigate these complex ethical considerations while upholding the principles of beneficence, autonomy, and non-maleficence.

Another important difference between children and adults is the duration of palliative care. In adults, the average survival time after the initiation of palliative care is 1–4 months [15], whereas in the paediatric population, the duration of palliative care hospitalisation ranges from hours to years. As paediatric palliative care often involves long-term support due to chronic illness or complex medical needs, it may require a continuum of care that extends into adulthood, requiring seamless transitions from paediatric to adult healthcare systems [13].

Overall, the combination of emotional challenges, complex medical conditions, close involvement with families, and the potential for loss can make working in paediatric palliative care more stressful than in other healthcare fields. However, it is important to note that many healthcare providers find great fulfilment in this work as they have the opportunity to make a significant positive impact on the lives of children and their families.

Burnout is a psychological syndrome characterised by emotional exhaustion, depersonalization, and a reduced sense of personal accomplishment [16]. In the context of paediatric palliative care, emotional exhaustion can result from constant exposure to the suffering and loss experienced by patients and their families. Witnessing children’s pain and distress can take a toll on the mental wellbeing of healthcare providers. In a pilot cross-sectional study, Kase et al. reported that emotional exhaustion, social isolation, and “recent involvement in a clinical situation in which no life-prolonging activities were initiated” were the most significant determinants of burnout among paediatric palliative care providers in the United States [17].

In a study of the emotional experiences of home-based paediatric palliative care workers, two main themes were identified: the change in workers’ lives for the better (positive changes in life priorities and goals, increased appreciation of life, improved personal resources and interpersonal relationships) [18,19] and also the negative effects of work (stressful and psychologically overwhelming work that leaves them with emotional trauma) [20].

Healthcare professionals are anxious and unprepared to talk about death and the end of life [21]. An online survey investigated the professional life of palliative care workers in Spain, focusing on the relationships between awareness, self-care, compassion satisfaction and fatigue, exhaustion, and coping with death. The results of this study provide an insight into the relationship between professionals’ awareness and their ability to cope with the end of life and death [22].

A study conducted in two hospitals in Korea examined differences in the moral distress among physicians and nurses providing end-of-life care. Most physicians and nurses experienced similar feelings of anger, helplessness, and burden due to the lack of adequate resources for end-of-life care. However, the factors and contexts of moral distress differed. Nurses referred mainly to the poor organisation of care, heavy workloads without support, and the provision of care without involvement in decision making. Doctors mentioned misconceptions about end-of-life care, the lack of communication due to fragmented hierarchies and disciplines, the burden of responsibility for making difficult decisions, and the pressure of allocating resources. The article pointed out the importance of mutual understanding through improved communication [23].

The complexity of palliative care is not only due to the nature of the patient and the identification of the family and the patient’s needs. It is compounded by the presence of many different “actors” involved in the care process, who may have different perceptions of the appropriateness of decisions [24]. All of this can lead to conflict between professionals and carers, or within the same multidisciplinary team, when the burden of responsibility for the decisions to be made is particularly heavy and especially when these decisions relate to the end of life [25].

A meta-analysis of the management of paediatric bereavement at the end of life highlighted the need for effective communication between health professionals and families, additional training for health professionals, improved symptom management planning including anticipatory management, and special attention to the patient’s perspective [26].

Palliative care workers face increasing burnout and a growing need for specialised services due to demographic changes and the increasing number of cases of serious, life-limiting illness. Moral distress occurs when health professionals want to do what they believe is right but are forced to act in a way they believe is morally wrong and against their core values. Moral injury occurs when a person’s “deeply held moral beliefs” are threatened by their actions or inactions. Moral injury can lead to burnout and resignation or have a negative impact on mental health [27].

Ethical decision-making challenges also arise in the care of children with chronic kidney disease who require dialysis and palliative care. In low- and middle-income countries, these children have limited access to maintenance haemodialysis, early kidney transplantation and palliative care, leading to difficult decision making and moral distress among healthcare workers [28].

Communication with children and their families is a key issue that can also be a major stressor for staff, and difficulties in this area can significantly influence the professional experience of palliative care workers. In our study, 26.8% reported difficulties in communicating with patients’ families. Quality communication is associated with the parents’ peace of mind, a sense of being acknowledged and comforted, and greater trust in the medical act [29]. Building strong relationships with patients and their families can be rewarding for palliative care workers and help reduce stress. Challenges include dealing with complex family dynamics and delivering bad news, which requires highly competent communication skills and emotional support [30]. A study on the experiences of nurses working in a community service showed the added complexity of caring for a child with both a life-limiting condition and an intellectual disability. Their dedication and commitment to providing high-quality, evidence-based care faces significant challenges and potential conflict when family members’ opinions differ from clinical judgment. These disagreements may be influenced by the emotional needs of parents in situations of intense stress [31].

The lack of resources, clinical workload, and time constraints are important issues in other studies: the lack of time spent with the child and family, the shortage of human resources, and late diagnosis were cited as reasons. Insufficient time for self-care and development has also been reported [32]. The difficulty in managing their emotions, the inability to perform their tasks within the expected time frames due to multitasking, the stress of being involved in communicating the diagnosis, and the feeling of helplessness as the child’s health deteriorates are challenges for nurses working in paediatric palliative care [33]. The death of children, especially after the withdrawal of life-sustaining therapies, is a complex process that leaves vulnerable families and the clinical team without adequate tools to support patients, families, and themselves. Knowing how to predict time to death could improve care and support for all involved [34].

A study assessed the wellbeing, burnout, and resilience of healthcare workers in the management of paediatric patients with neurological pathology. Moreover, 47% of the workers experienced high levels of burnout. The observation of progressive symptoms until death and involvement in emotionally charged interactions revealed that workers felt helpless and unsupported at work [35].

COVID-19 pandemic restrictions have had a significant impact on the work of paediatric palliative care staff, creating a number of challenges and increasing professional stress. The emotional wellbeing of patients, families, and hospice care staff was affected [36] as were changes in workplace protocols and reduced social interaction [37]. Extra-organisational factors and uncertainty about the future, manifested as anticipatory anxiety, had a higher impact on workers’ wellbeing than in non-pandemic conditions [38]. The therapeutic protocol was difficult to implement because it had to take into account COVID-19 procedures, which were dynamically modified and applied to patients and staff. The use of full protective equipment and other adjustments to working conditions were perceived as an additional burden [39]. In the context of the COVID-19 pandemic, the impairment of work capacity due to employee illness had a negative impact on professional activity. Adapting working hours, temporarily eliminating night shifts, and adjusting circadian rhythms improved workers’ quality of life [40]. In addition, post-COVID syndrome has caused difficulties in completing tasks and maintaining work schedules due to long-term symptoms, in some cases leading to temporary measures to adapt the workplace to allow workers to recover [41]. Another stress factor for healthcare workers has been the fear of becoming contaminated by the patients they care for and subsequently developing a variety of infectious pathologies, either as a result of decreased resistance due to overwork or as a complication of pre-existing chronic conditions [42,43,44,45].

Notable stressors included changes in team organisation, the absence of children’s family members, concerns about personal and colleagues’ health, and fears about the financial sustainability of health programmes [46,47]. The mental health of palliative care workers was significantly affected, with high levels of stress, anxiety, and depression reported, particularly among female, younger, or non-religious staff [48].

Preventing stress and burnout among healthcare workers, particularly in paediatric palliative care, requires a multifaceted approach. A variety of methods and interventions can be implemented at the organizational level, such as the use of counselling and psychosocial support programmes for employees, which may include individual therapy sessions, support groups, and workshops on managing emotions and stress [49]. Palliative care workers, unlike other clinical specialties, face unique stressors related to ongoing exposure to pain, suffering, and death. Organisational support and training in coping strategies are essential to reduce burnout [50]. A qualitative study of the factors that influenced nurses’ decisions to work in palliative care and their attitudes towards life and work found high levels of commitment, meaning, and purpose in their work. One area of variation was responsiveness to change, which was closely related to their training and consistency. Staff motivation, training, and support can have a positive impact on the quality of patient care. The importance of resilience and wellbeing at work is reflected in similar levels of psychological distress but lower levels of burnout in palliative care workers compared to other specialties [51].

There is an acute shortage of specialists and a lack of dedicated services for paediatric palliative care in Romania. In 2020, the situation was characterised by the existence of isolated paediatric palliative care services without nationwide coverage. Although the need for children’s palliative care is evident, the number of qualified workers in this field is very low in relation to the demand [52]. This adds to the burden on the few existing professionals and may lead to increased stress and burnout among them.

The availability of palliative care services for children varies across Europe. In Romania, a country with one of the lowest healthcare expenditures in Europe, palliative care is mainly provided by charitable organisations. There is a limited amount of literature and research on paediatric palliative care in Romania. A 2021 study conducted four mixed focus groups in four university paediatric oncology centres in three different regions of Romania (Bucharest–Ilfov, North-East and North-West). For many workers, the emotional burden of work, unhealthy work–life balance, and staff shortages were among the main barriers to the successful integration of paediatric palliative care. Staff shortages were attributed to a lack of financial resources and the continuing cultural stigma associated with palliative care and oncology. Political unrest was also identified as a major barrier to the implementation of palliative care [53].

Identifying sources of work-related stress and rewards emphasised the need to manage staff distress and prevent long-term burnout [54] and to implement counselling or training programs to help healthcare workers [55]. Another study highlights organisational interventions that could reduce stress and burnout in neonatal intensive care units, with potential implications for paediatric palliative care [56] and institutional staff engagement [57].

A project to improve the quality of care in a paediatric teaching hospital used a case study and a simulation-based educational programme to reduce anxiety in graduate nurses working in end-of-life care. Anxiety levels decreased by 24.1% after participation in the program [58]. A synthesis of the importance of clinical supervision as a method of reducing nurse stress and burnout and the practice of clinical supervision for paediatric palliative care nurses showed that it was a method of reducing nurse stress and exhaustion [59].

The use of simulation-based learning in nurse education increases learning capacity, the understanding of the importance of teamwork, and interdisciplinarity, which is a difficult but crucial part of palliative care practice. Personal growth has also been observed after participation in this learning model, which can be an important protective factor against stress. Emphasis should be placed on more practical content and clinical and ethical skills [60], including training to work under special conditions (epidemics and pandemics) [61].

The aim of this article is to shed light on the multifaceted aspects of stress factors in paediatric palliative care, providing information on its causes, consequences, and possible mitigation strategies in the specific context of the north-east region of Romania, as there is a clear lack of research in this area, which is still in its early stages of development in our country. By understanding the contributing factors and exploring effective interventions, organisations and healthcare providers can work together to create a supportive and sustainable environment that promotes the wellbeing of healthcare workers and improves the care offered to vulnerable patients and their families.

## 2. Materials and Methods

We conducted a qualitative cross-sectional study to assess the main sources of stress among palliative care staff, the impact of the COVID-19 pandemic on stress levels, and the emotional impact in paediatric palliative care compared to adult palliative care and to identify the resources and support needed to maintain and improve the wellbeing of paediatric palliative care staff.

Participants: The study group participants were professionals working in the palliative care unit of the “Sf. Maria” Emergency Children’s Hospital in Iasi, the Lumina Association–Children’s Palliative Care Centre in Bacau. We also sent the questionnaire to other professionals from palliative care wards and hospice care units in North-East Romania who had worked in the field of paediatric palliative care at some point in their career and were working in adult palliative care at the time of our study, regardless of their specialisation (doctors, nurses, psychologists, social workers, physiotherapists, spiritual counsellors, and other categories directly involved in palliative care services).

Study inclusion criteria: healthcare professionals working in paediatric palliative care and adult palliative care with experience in the field, regardless of their level of experience and working in a variety of work settings, including hospitals, clinics, palliative care centres, and other medical institutions.

Exclusion criteria: people not involved in any activity or field related to palliative care, people not employed in the health or medical sector, and participants who did not complete the questionnaire.

For the selection of the study group, we used a non-probability sampling method, a type of convenience sampling based on specific predetermined criteria. By applying the inclusion and exclusion criteria, we aimed to obtain a representative and appropriate sample for our study. We also used the direct invitation sampling method by sending a link to the online questionnaire. This approach was helpful in identifying and recruiting those participants who met the selection criteria and who were interested and available to attend the study.

We used a 12-question survey. We developed a questionnaire based on discussions with healthcare workers who were examined at regular occupational health check-ups, according to their needs, feedback, and job description, to make an inventory of stressors experienced by paediatric palliative care workers. We designed the survey taking into account the questions in similar studies in the literature and tested it first on a small number of 6 workers to see if the questions were understood, clear, and could be discussed.

We developed our questionnaire based on our knowledge of the Romanian context [52,53] and the results of the studies in the literature on the causes and effects of stress perceived by palliative healthcare professionals at work [4,9,14,17,18,19,20,22,23,24,27,28,30,39,46] (Table 1), the impact of the COVID-19 pandemic on stress and burnout in palliative care [36,37,39,46,47,48], and expectations and solutions for improving the workplace [4,14,18,34,35,36,49,50,55,57,58,59]. All this formed the theoretical basis of this study and contributed to the identification of the issues, the development of the hypothesis and the items of the questionnaire, and the overall aim of the research. The use of this theoretical framework facilitated the development of a scientifically based approach to investigating the stress and needs of healthcare professionals working in this field.

We identified the categories and subcategories of questions to be included in the questionnaire to comprehensively address stress and burnout in palliative care workers. We carefully considered how to phrase the questions to make them as clear and relevant as possible for those surveyed. We also considered psychometric principles to ensure the validity and reliability of the questionnaire.

We pre-tested the survey with a small group of palliative care professionals to ensure that it was appropriate and understood by respondents. This process allowed us to identify any difficulties in understanding the questions or any ambiguities, and the questionnaire was adjusted accordingly.

Both multiple-choice and open-ended questions were included to encourage respondents to provide comprehensive and contextual information. We preferred a reduced-item questionnaire to encourage participants to answer honestly and to the point and to avoid biased responses and withdrawals from the study.

The questionnaire has not been validated. Since Romania is at the beginning of the development of paediatric palliative care and there are few specialists working in this field, we have a starting point for the elaboration of tools to study stressors and solutions for their management.

We distributed the questionnaire via an online link to healthcare workers. They were informed about the purpose of the study, the confidentiality of their data, and how to answer the questionnaire voluntarily and anonymously.

The data were collected in November 2023. Each completed questionnaire was recorded and stored in a secure database for further analysis. The collected data were processed and statistically analysed to identify relevant trends, correlations, and patterns in the responses of palliative care professionals. Our aim was to gain meaningful insights into the causes of stress and burnout, the impact of the pandemic, and the needs of paediatric palliative care workers in North-East Romania. As the field and the training of professionals are still developing in our country, this study is a starting point for further interventions and initiatives.

## 3. Results

**Figure 1:**
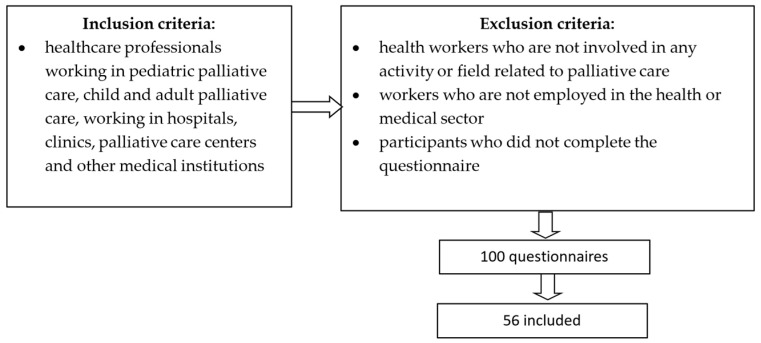
Inclusion and exclusion criteria of the study group.

We distributed 100 questionnaires, of which 57% were returned. Of these, we rejected one questionnaire during data analysis because it was returned incomplete (Figure 1).

In this study, we grouped and analysed the results into three distinct sections to better understand the experiences and needs of palliative care staff.

In the first section, we analysed demographic data to provide an overview of the study participants. This information included age, gender, job category, and the type of organisation in which they worked.

In the second part, we explored the causes and consequences of stress experienced by healthcare professionals at work. Questions related to the main sources of stress, such as workload, dealing with children’s distress, losing patients, and communicating with families. Another important topic was the impact of the COVID-19 pandemic on levels of stress and burnout in the context of increased workload. This section provided a detailed picture of specific stressors in paediatric palliative care and their impact on the medical team.

In the third category, we analysed the expectations and solutions proposed by healthcare professionals to improve job satisfaction in paediatric palliative care. The participants were asked to identify the resources and support they considered essential to cope with the specific stressors in this area. We also assessed their solutions and suggestions for how organisations can help to optimise working conditions and support professional wellbeing.

### 3.1. Demographic Data

In total, fifty-six palliative care workers participated in the study, comprising 53 women (96.4%) and 2 men (3.6%).

The mean age was 45.85 years, with a median of 47.5 years.

The distribution by age group was 21–30 years (3.57%), 31–40 years (21.43%), 41–50 years (41.07%), 51–60 years (32.14%), and over 61 years (1.79%).

In total, 68.5% of respondents worked in adult palliative care services, 20.4% in paediatric palliative care, and 11.1% in both adult and paediatric palliative care.

Of the professional categories that participated in the survey, 7.1% were doctors and 73.2% were nurses, and others included social workers (3.6%), physiotherapists (1.8%), psychologists (1.8%), chaplains (1.8%), and care coordinators (1.8%).

### 3.2. Causes and Effects of Stress Perceived by Healthcare Professionals at Work

**Figure 2:**
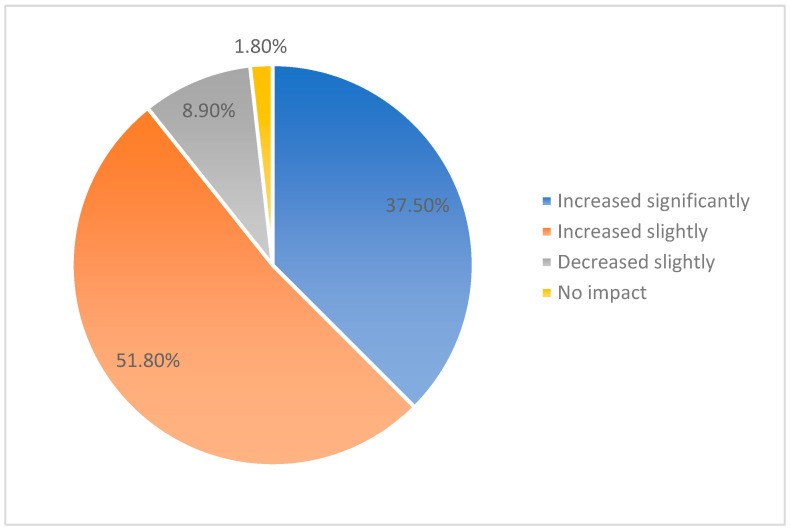
Impact of the COVID-19 pandemic on stress and burnout levels in palliative care workers.

- There were many responses to the question “*What do you consider to be the main causes of stress in your work in palliative care?*”
- High workload (57.1%) is considered by staff to be one of the main causes of stress in palliative care. The time and effort required to care for patients is higher in order to ensure the quality of care.
- Moreover, 55.4% of respondents said that the difficult emotional burden of dealing with the suffering of patients and their families is a significant cause of stress.
- In total, 6.4% of respondents said that the death of a patient has a profound emotional impact that requires adequate support to cope with the loss.
- Furthermore, 26.8% of professionals reported that dialogue/communication with patients’ families and managing relationships with them was a significant source of stress. Additionally, 1.8% of participants mentioned the difficulty of working with doctors, patients’, and carers’ expectations and bureaucracy, administrative workload, and inadequate pay.
- In response to the question “*How has the COVID-19 pandemic affected your level of stress and exhaustion?*”, 51.8% of the survey group said that their level of stress and exhaustion had increased significantly in relation to the pandemic due to increased pressure on the healthcare system and challenges related to personal safety and managing the risk of infection.
- Moreover, 37.5% of respondents reported that their levels of stress and fatigue had been slightly affected. Even if they did not experience a significant impact, the pandemic still had a negative effect on their psychological wellbeing and level of exhaustion.
- Moreover, 1.8% of participants reported that their levels of stress and fatigue had decreased slightly because they had found ways to adapt and manage stress.
- Furthermore, 8.9% of respondents reported that the pandemic had no effect on their levels of stress and exhaustion. This category of respondents may represent professionals who already had effective stress management strategies in place or had adequate resources and support (Figure 2).

The Spearman rank correlation analysis between the age of the respondents and the change in stress levels due to the COVID-19 pandemic shows a correlation coefficient of about 0.302. This indicates a moderate positive correlation between the age of the respondents and the degree to which their stress levels changed due to the pandemic: as the age of the respondents increases, there tends to be a slight increase in the magnitude of the change in stress they report, whether it is an increase or decrease in stress levels. This moderate correlation suggests that age may play a role in how individuals experienced changes in stress levels due to the pandemic, but it is not a strong predictor on its own.

- In response to the question “*How do you think paediatric palliative care differs from adult palliative care in terms of emotional impact and stress?*”, 40.7% of respondents felt that there were differences in communication with patients and their families. Dealing with children and their parents involves complex, sensitive issues and making difficult decisions in a way that is appropriate to the child’s age and development.
- In total, 72.2% of respondents cited special challenges related to children’s needs as a major stressor. Children have complex medical needs and can experience high levels of distress that are difficult to manage.
- Moreover, 74.1% of paediatric palliative care professionals feel a strong emotional connection with the children they care for and their families, with profound satisfactions as well as significant emotional demands.

The causes of perceived stress at work are summarised in Table 2.

**Figure 3:**
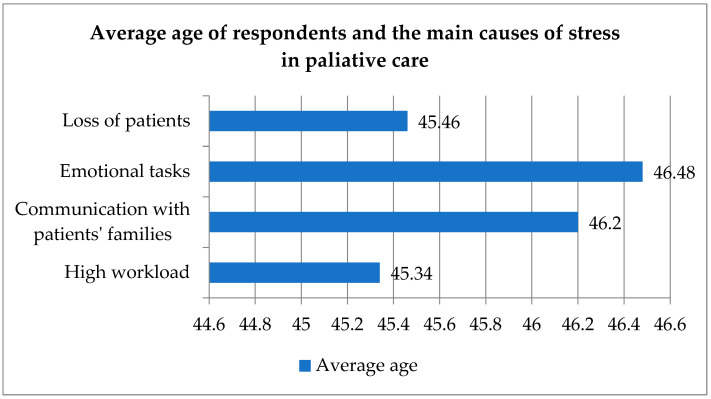
Average age of respondents and the main causes of stress in palliative care workers.

These results suggest slight differences in the average age of respondents according to the main sources of stress they reported (Figure 3). There is not necessarily a direct correlation between age and specific causes of stress but rather provides an initial exploration of possible patterns.

**Figure 4:**
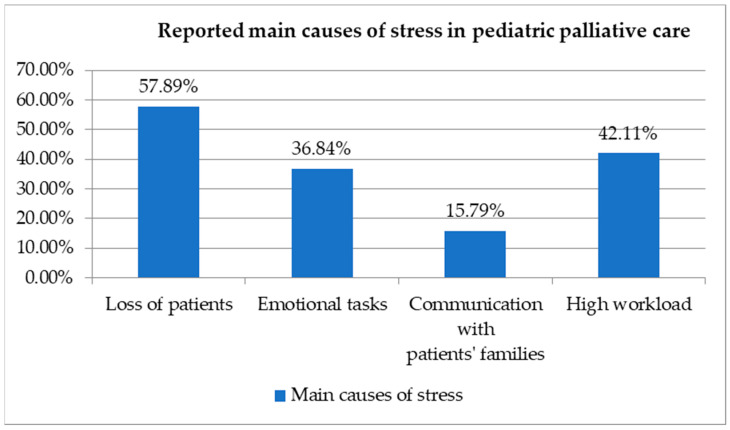
Reported main causes of stress among paediatric palliative care workers.

These results suggest that losing patients and high workloads are the most commonly reported causes of stress among those working in paediatric palliative care (Figure 4). Emotional tasks are also a significant stressor, although to a lesser extent, while communication with patients’ families is less frequently cited as a major source of stress in this specific care setting. This analysis provides insight into the unique challenges faced by healthcare professionals in paediatric palliative care and highlights areas where additional support and resources may be helpful.

### 3.3. Expectations and Solutions for Improving Wellbeing at Work

- In response to the question “*What are your main expectations of your workplace in terms of supporting and improving wellbeing?*”, 53.6% of respondents said that a key expectation was access to training and professional development programmes to improve their skills and keep up with new developments in palliative care. Additionally, 32.1% of workers cited the need for access to psychological counselling, 37.5% the need for emotional support, 51.8% the need for more recognition and appreciation of their work, and 1.8% the need for more respect, honesty, and tolerance.
- When asked “*How do you think you can be better helped to cope with difficult work situations?*”, 48.2% of employees cited support from colleagues and teamwork, 46.4% considered access to stress management resources important, and 39.3% liked flexibility in working hours. In total, 33.9% said that constructive feedback and regular professional guidance can help them overcome challenging moments at work. Moreover, 5.4% mentioned other means of support: a balanced organisational climate based on trust and cooperation.
- The open-ended question about additional suggestions for improving working conditions and reducing stress in palliative care had a wide range of responses. These included the following: promoting teamwork, organising team-building activities, increasing the number of vacation days, and providing regular debriefing sessions. This allows professionals to discuss difficult experiences and express their feelings.

Other solutions suggested include the need to improve infrastructure (adequate equipment and facilities), the recruitment and retention of sufficient staff, the importance of fair and equitable remuneration of palliative care professionals, and reducing workload by limiting the number of patients assigned to each worker.

The provision of free specialist training can contribute to the continuous development of palliative care professionals’ knowledge and skills and increase their confidence in their own abilities.

- In response to the question “*What resources and support do you think would be essential to cope with specific work pressures in pediatric palliative care?*”, 54% of workers felt that training in paediatric palliative care was essential to deal with the specific stress. In total, 46% believed that support groups and peer discussions are valuable resources for stress management, 40% would like to receive specialised psychological support, and 60% considered it was essential to implement organisational policies and practices that take into account the specificities and work challenges of paediatric palliative care as well as their personal health and wellbeing.

The expectations and solutions for optimising wellbeing at work are summarised in Table 3.

## 4. Discussion

The results of the questionnaire reflect significant opportunities for the development and implementation of support tools in paediatric palliative care in the north-east region of Romania, given the early stage of this specialty in our country. These results can be a starting point for identifying and addressing the needs of health professionals involved in the care of children with chronic progressive diseases and the support of their families.

Of the participants interviewed in our study, 20.4% reported working in paediatric palliative care services and 11.1% worked in both adult and paediatric palliative care services. These findings reflect the current situation in Romania, where services for children with progressive chronic illnesses reaching the terminal stage are isolated and insufficient compared to the existing needs [48,49]. The low number of qualified specialists in paediatric palliative care and the limited availability of services dedicated to children is a major problem in the Romanian healthcare system. This situation places an additional burden on the medical staff working in these services as they have to manage the complex needs of children and their families with limited resources [48,49].

In our study group of paediatric palliative care, there are significant differences between the practice of nurses working continuously at the patient’s bedside and that of the paediatric palliative care specialist doctors. In Romania, physicians specializing in paediatric palliative care are very few in number and are generally involved in consultative and care coordination roles, providing expertise and support in pain management, symptom management, and the development of personalised care plans. They are not involved in on-call activities, which can be a challenge to effectively manage emergencies or complex care needs outside of normal working hours [52,53].

In our study group, the representative age range was 41–60 years and female, which is the predominant group in healthcare and particularly in palliative care.

The identification of high levels of emotional involvement (74.1%) and specific challenges related to grief management in children (72.2%) in our results highlights that paediatric palliative care staff face significant emotional involvement and specific challenges in managing children’s distress, which is consistent with previous experience and knowledge in the field [17,18,19,20,25,27,35].

The finding that a high workload (57.1%) was considered one of the main causes of stress highlights the high pressures and demands associated with working in paediatric palliative care in Romania [52,53], where professionals are often faced with multiple and complex tasks, tight deadlines, and a high volume of responsibilities. Physical exhaustion has been associated with a dynamic and prolonged duration of care [4,23,32,33].

Moreover, 55.4% of respondents identified emotionally difficult tasks as one of the main sources of stress in their work in paediatric palliative care. This is in line with the complexity and sensitivity of the field and with other authors who have analysed the emotional difficulties [31,35,36,49] and moral distress in paediatric palliative care [23,27,28].

In total, 46.4% of respondents cited the death of a patient as a major source of stress in their work in paediatric palliative care, emphasising the profound emotional impact of loss in this sensitive area [20,22,34].

The fact that 36.7% of respondents believe that working in paediatric palliative care affects their emotional balance and 12.2% report that it affects their personal life, including relationships and family life, while 61.2% say it influences their own outlook on life, shows the multiple dimensions of stress and burnout in this field. The impact on psychological stability may be the result of exposure to patient suffering and pain [17,46,48,49], difficult emotional situations [20,25,27,31,35,36,49], and the pressure to provide high-quality care [28,29,38,39]. The effects on personal life can be related to the high demands of work, which can lead to a lack of time and energy for family and personal relationships. In addition, the influence on life perspective may be reflected in the way professionals perceive their own lives and the meaning of their work in the context of their professional experiences [16,18,19,20].

Because of the constant exposure to patient and family suffering, paediatric palliative care providers are at risk of developing compassion fatigue [18,22]. Physical exhaustion, personal history of trauma, and the internalisation of stressful issues were factors that directly influenced wellbeing and work performance [17]. Change for the better and personal growth also occur in the lives of paediatric palliative care workers. A thorough understanding of the phenomenon of personal growth could help organisations to implement innovative approaches that counteract compassion fatigue, thereby improving both staff satisfaction and child care performance [18].

In total, 51.8% of the survey group reported that their levels of stress and exhaustion had increased significantly in the context of the pandemic due to the enhanced pressure on the healthcare system and challenges related to personal safety and managing the risk of infection. This is consistent with the literature. Causes of moral distress related to the pandemic included patient-related factors (patients not receiving requested treatments due to shortages), healthcare worker-related factors (the tension between working in the healthcare system and the possibility of infecting loved ones, sickness, and absence from work), institutional factors (delays in treatment), and public health policy and directive-related factors (the impact of government policy directives on an individual’s ability to work) [36,37,39,40,41,44,46,47].

The expectations and solutions for improving the wellbeing of the palliative care workers who participated in our study were diverse and resulted in multiple responses to the open-ended question, demonstrating the desire of these professionals to work in optimal conditions.

The respondents to our survey indicated that access to education and training programmes was a key expectation, which is consistent with findings in the literature that emphasise the importance of continuing medical education to improve skills, keep up with new developments in palliative care, and increase confidence in one’s own abilities [58,60,61].

Workers in our study group mentioned the need for access to psychological counselling (32.1%), emotional support (37.5%), more recognition and appreciation of their work (51.8%), more respect, honesty and tolerance (1.8%), support from colleagues and team collaboration (48.2%), and access to stress management resources (46.4%). Moreover, 33.9% said that constructive feedback and regular professional guidance can help them to overcome challenging moments at work.

Our respondents suggested technical and organisational measures to optimise the work process: the flexibility of working hours, the need to improve infrastructure (adequate equipment and facilities), the recruitment and retention of sufficient staff, the importance of fair and equitable remuneration of palliative care professionals, and workload reduction by limiting the number of patients assigned to each worker.

## 5. Conclusions

The study provides an opportunity to highlight the needs and challenges of staff involved in paediatric palliative care compared to adult palliative care at a time when the field is still developing in Romania.

Our study shows that stress in paediatric palliative care is a complex problem influenced by several factors: the emotional burden of care (the emotional support of patients and families), difficult ethical and moral decisions about life-prolonging treatments, and discussions about prognosis and care decisions with families and children. Working with different medical specialties and professions in palliative care can be complicated and lead to interpersonal tensions. Staff often become emotionally attached to patients and their families, and the repeated deaths of patients can cause significant emotional distress. The desire to provide the best possible care and to meet the expectations of families and patients can put additional pressure on staff.

Paediatric palliative care is physically and emotionally demanding, resources are limited, and time pressures, administrative tasks, and the lack of organisational support can contribute to professional stress. The nature of the work can make it difficult for staff to maintain a healthy work–life balance. The COVID-19 pandemic has had a profound impact on the stress levels and psychological health of paediatric palliative care staff, with changes in service delivery, increased work-related distress, and emotional strain being prevalent. The expectations of paediatric palliative care professionals in terms of supporting and improving wellbeing in the workplace are complex and varied. They include the following: training programmes, psychological counselling, supportive work environment, and access to stress management resources.

The results of this study can be used to inform policy makers, healthcare managers, and other stakeholders about the need and importance of developing and strengthening paediatric palliative care services in Romania and other countries with similar problems.

## 6. Study Limitations

Our study had a relatively small cohort of 56 palliative care professionals, of whom 20.4% worked exclusively in paediatric palliative care and 11.1% in mixed adult and paediatric palliative care services. This may affect the representativeness of the outcomes and their extrapolation to the whole population of professionals in the field. The results of our study highlight the need and urgency to improve access to quality paediatric palliative care in North-East Romania and underline the importance of strengthening the capacity to care for and support terminally ill children and their families. In the future, we plan to work together with other paediatric palliative care centres in the country to obtain an overview of these services on a national level.

The study used an online questionnaire to collect data. The participants who completed the survey may have been those who were more motivated or interested in the topic, which may have influenced the results. The questionnaire was short. This was carried out to encourage participants to be honest and to the point.

Responses to questions about emotional impact and stress may be influenced by the subjects’ perceptions of their own condition. Professionals may underestimate or overestimate the impact of stress or their work on themselves. Open-ended responses may be subjective and difficult to quantify or compare objectively. The interpretation of these responses may vary according to the researcher’s interpretation.

The study did not control for or account for some important variables that may influence emotional impact and stress in paediatric palliative care, such as previous work experience or specific organisational resources. Workers who took part in the study may have different characteristics or experiences that could influence the results. For example, those who have had worse experiences may be more motivated to participate.

Future research should have a larger pool of participants and control for key variables, use a mixed methods research approach to obtain different perspectives, and consider the specifics of each palliative care setting to obtain more generalisable results.

## Figures and Tables

**Table 1:** Elements of the questionnaire and scientific literature used to develop it.

Study Purpose	Elements from the Questionnaire	Specific References
Assessment of stress and burnout in paediatric palliative care	- Increased emotional involvement in childcare - Managing children’s suffering - High workload - Emotionally difficult tasks - Influencing personal perspective on life - Patients’ death - Communicating with children’s family - Impact on emotional balance - Impacts on family life	Rico-Mena et al. (2023) [4] Blazin et al. (2018) [9] Saad et al. (2022) [14] Kase et al. (2019) [17] Beaune et al. (2018) [18] Pereira et al. (2023) [27] Sansó et al. (2015) [22]
Impact of COVID-19 on stress and burnout in palliative care	- Stress and exhaustion levels in the COVID-19 pandemic	Kates et al. (2021) [36] Feeley et al. (2021) [38] Vig et al. (2022) [46] Bradshaw et al. (2022) [48] Chan et al. (2022) [49]
Expectations and solutions regarding the optimization of workplace wellbeing in paediatric palliative care	- Training and development programs - Valuation and appreciation - Co-workers support - Access to stress management resources - Flexible working schedules - Support in tasks management - Positive feedback and periodic evaluation - Access to psychological counselling support	Rico-Mena et al. (2023) [4] Saad et al. (2022) [14] Sansó et al. (2015) [22] Pereira et al. (2023) [27] Green et al. (2020) [57] Beavis et al. (2021) [59]

**Table 2:** Causes and consequences of healthcare workers’ perceptions of work stress by number of responses.

Causes of Perceived Stress at Work	Number of Responses	Percent
Increased emotional involvement in childcare	40	74.1%
Managing children’s suffering	39	72.2%
High workload	32	57.1%
Emotionally difficult tasks	31	55.4%
Influencing personal perspective on life	30	61.2%
Patients’ death	26	46.4%
Communicating with children’s family	22	26.8%
Impact on emotional balance	18	36.7%
Impacts on family life	6	12.2%
Other	12	10%

**Table 3:** Expectations and solutions for improving wellbeing at work by number of responses.

Expectations and Solutions for Optimising Wellbeing at Work	Number of Responses	Percent
Training and development programs	30	53.6%
Valuation and appreciation	29	51.8%
Co-workers support	27	48.2%
Access to stress management resources	26	46.4%
Flexible working hours	22	39.3%
Tasks management support	21	37.5%
Positive feedback and regular appraisal	19	33.9%
Access to psychological counselling support	18	32.1%
Other	6	6%

## Data Availability

All data are available from the first author and the corresponding author.
